# Tacrolimus Exposure Before and After a Switch From Twice-Daily Immediate-Release to Once-Daily Prolonged Release Tacrolimus: The ENVARSWITCH Study

**DOI:** 10.3389/ti.2023.11366

**Published:** 2023-08-01

**Authors:** Caroline Monchaud, Jean-Baptiste Woillard, Sabrina Crépin, Naïma Tafzi, Ludovic Micallef, Jean-Philippe Rerolle, Sébastien Dharancy, Filomena Conti, Gabriel Choukroun, Antoine Thierry, Matthias Buchler, Ephrem Salamé, Cyril Garrouste, Christophe Duvoux, Charlotte Colosio, Pierre Merville, Dany Anglicheau, Isabelle Etienne, Faouzi Saliba, Christophe Mariat, Marilyne Debette-Gratien, Pierre Marquet

**Affiliations:** ^1^ Department of Pharmacology, Toxicology and Pharmacovigilance, Centre Hospitalier Universitaire de Limoges, Limoges, France; ^2^ INSERM1248 Pharmacolgy and Transplantation, Limoges, France; ^3^ Fédération Hospitalo-Universitaire Survival Optimization in Organ Transplantation (FHU SUPORT), Limoges, France; ^4^ Unité de Vigilance des Essais Cliniques, Centre Hospitalier Universitaire de Limoges, Limoges, France; ^5^ Department of Nephrology, Dialysis and Transplantation, Centre Hospitalier Universitaire de Limoges, Limoges, France; ^6^ Department of Hepatology-Transplantation, CHU Lille, Lille, France; ^7^ Department of Hepato-Gastro-Enterology, Hôpital Pitié-Salpêtrière, Paris, France; ^8^ Department of Nephrology, Internal Medicine, Transplantation, Centre Hospitalier Universitaire (CHU) d'Amiens, Amiens, France; ^9^ Fédération Hospitalo-Universitaire Survival Optimization in Organ Transplantation (FHU SUPORT), Poitiers, France; ^10^ Department of Nephrology, Hemodialysis and Renal Transplantation, Centre Hospitalier Universitaire (CHU) de Poitiers, Poitiers, France; ^11^ Fédération Hospitalo-Universitaire Survival Optimization in Organ Transplantation (FHU SUPORT), Tours, France; ^12^ Department of Nephrology–Arterial Hypertension, Dialyses, Renal Transplantation, Centre Hospitalier Universitaire de Tours, Tours, France; ^13^ Center for Hepatobiliary and Pancreatic Surgery, Hepatic Transplantation, Centre Hospitalier Universitaire de Tours, Tours, France; ^14^ Department of Nephrology–Hemodialyses, Centre Hospitalier Universitaire de Clermont-Ferrand, Clermont-Ferrand, France; ^15^ Department of Hepatology, Hôpital Henri-Mondor, Assistance Publique Hôpitaux de Paris, Créteil, France; ^16^ Department of Nephrology, Centre Hospitalier Universitaire de Reims, Reims, France; ^17^ Department of Nephrology, Transplantation, Dialysis and Aphereses, Centre Hospitalier Universitaire de Bordeaux, Bordeaux, France; ^18^ Department of Kidney and Metabolism Diseases, Transplantation and Clinical Immunology, Hôpital Necker-Enfants Malades, Paris, France; ^19^ Department of Nephrology, Hemodialysis, Transplantation, Centre Hospitalier Universitaire (CHU) de Rouen, Rouen, France; ^20^ Hôpital Paul Brousse, Villejuif, France; ^21^ Department of Nephrology, Dialysis and Renal Transplantation, Centre Hospitalier Universitaire (CHU) de Saint-Étienne, Saint-Etienne, France; ^22^ Department of Hepato-Gastro-Enterology and Nutrition, Centre Hospitalier Universitaire de Limoges, Limoges, France

**Keywords:** kidney transplantation, liver transplantation, LCP-tacrolimus, AUC monitoring, dried blood spots, conversion, therapeutic drug monitoring, dose individualization

## Abstract

LCP-tacrolimus displays enhanced oral bioavailability compared to immediate-release (IR-) tacrolimus. The ENVARSWITCH study aimed to compare tacrolimus AUC_0–24 h_ in stable kidney (KTR) and liver transplant recipients (LTR) on IR-tacrolimus converted to LCP-tacrolimus, in order to re-evaluate the 1:0.7 dose ratio recommended in the context of a switch and the efficiency of the subsequent dose adjustment. Tacrolimus AUC_0–24 h_ was obtained by Bayesian estimation based on three concentrations measured in dried blood spots before (V2), after the switch (V3), and after LCP-tacrolimus dose adjustment intended to reach the pre-switch AUC_0–24 h_ (V4). AUC_0–24 h_ estimates and distributions were compared using the bioequivalence rule for narrow therapeutic range drugs (Westlake 90% CI within 0.90–1.11). Fifty-three KTR and 48 LTR completed the study with no major deviation. AUC_0–24 h_ bioequivalence was met in the entire population and in KTR between V2 and V4 and between V2 and V3. In LTR, the Westlake 90% CI was close to the acceptance limits between V2 and V4 (90% CI = [0.96–1.14]) and between V2 and V3 (90% CI = [0.96–1.15]). The 1:0.7 dose ratio is convenient for KTR but may be adjusted individually for LTR. The combination of DBS and Bayesian estimation for tacrolimus dose adjustment may help with reaching appropriate exposure to tacrolimus rapidly after a switch.

## Graphical Abstract

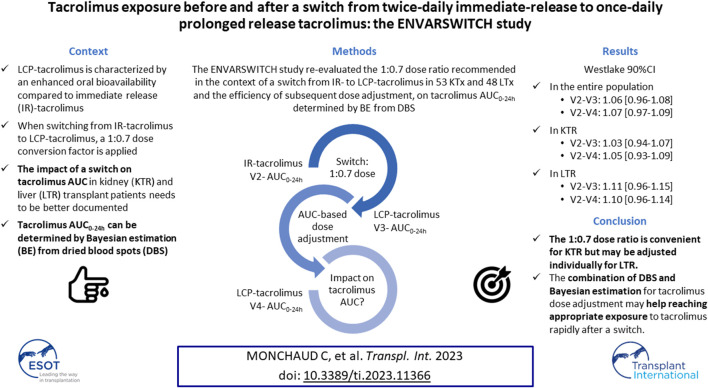

## Introduction

The pharmacokinetics of LCP-tacrolimus (Envarsus^®^) has been sparsely investigated [1], and clinical trials [2–5] have left some uncertainty on the exact starting dose, dose ratio with regards to other prolonged-release formulations, and blood levels to be expected in kidney (KTR) and liver transplant recipients (LTR). Previous experience with Advagraf^®^ showed that absorption could be almost nil in the first days post-transplantation, and that in stable patients, the 1:1 dose ratio resulted in lower C_0_ but comparable AUC_0–24 h_ [6].

The relationship between tacrolimus exposure and effects renders individual dose adjustment essential to avoid under- or overexposure [7]. The exposure index best associated with clinical effects is the area under the concentration–time curve (AUC) [7]. To overcome the inconveniences of collecting 10–12 blood samples over the dose interval, Bayesian estimators based on sparse sampling strategies have been developed for the AUC estimation of all tacrolimus formulations [1, 8–12] and are routinely used through the ISBA expert system^1^ [13]. However, the collection of several blood samples by venipuncture in a medical environment induces costs and logistical constraints. Therefore, dried blood spot (DBS) sampling, which can easily be performed at home, has been proposed for the therapeutic drug monitoring (TDM) of tacrolimus [14–20]. After a fingerprick, blood is applied onto a special filter paper, which is subsequently mailed to the laboratory. Good acceptability by the patients [21] and reliability of measured drug levels [16–20] are arguments in favor of DBS for the TDM of tacrolimus in transplantation. Furthermore, DBS are particularly suited to LCP-tacrolimus for which the optimal sampling times for AUC_0–24 h_ estimation are 0, 8, and 12 h post-dose [1].

In this context, we hypothesized that implementing DBS home sampling for the Bayesian estimation of tacrolimus AUC_0–24 h_ before and after a conversion, and considering the pre-switch AUC_0–24 h_ as a reference for LCP-tacrolimus dose adjustment after the switch, would allow maintaining of tacrolimus AUC_0–24 h._ Therefore, the aims of the ENVARSWITCH study were to verify, in KTR and LTR, the equivalence of the AUC_0–24 h_ values before and after a switch from IR-tacrolimus to LCP-tacrolimus at a 1:0.7 dose, followed by individual dose adjustment targeting the pre-switch AUC_0–24 h_. The study also aimed to compare tacrolimus exposure indices (AUC_0–24 h_, C_max_ and C_0_) before vs. after the switch, before and after dose adjustment.

## Patients and Methods

### Study Design, Patients and Procedures

The ENVARSWITCH study (EudraCT number: 2016-001014-22) was a multicenter prospective open clinical study conducted in 16 French transplantation centres, in accordance with the Declaration of Helsinki, Good Clinical Practice and the International Conference on Harmonization (ICH) guidelines. The protocol received approval from the Independent Ethics Committee (ref. CPP16-022/2016-001014-22) and authorization from the French National Agency for Medicines and Health Products Safety (ref. 160372A-11)*.* All enrolled patients gave their written informed consent.

The primary objective was to verify the absence of difference between pre- and post-switch tacrolimus AUC_0–24 h_ calculated by Bayesian estimation, in KTR and LTR switched from IR-tacrolimus (Prograf^®^) to LCP-tacrolimus (Envarsus^®^) at a 1:0.7 dose, possibly followed by individual dose adjustment targeting the pre-switch AUC_0–24 h_.

We enrolled adult (≥18 year-old) kidney and liver transplant recipients, transplanted for between 2 weeks and 1 year, in whom a switch from IR-tacrolimus to LCP-tacrolimus had been decided, and in whom the IR-tacrolimus dose had been unchanged for at least 1 week or since the last two C_0_ measurements. At the first protocol visit (V1), tacrolimus C_0_ had to be between 4 and 12 μg/L and hematocrit >0.27.

**FIGURE 1:**
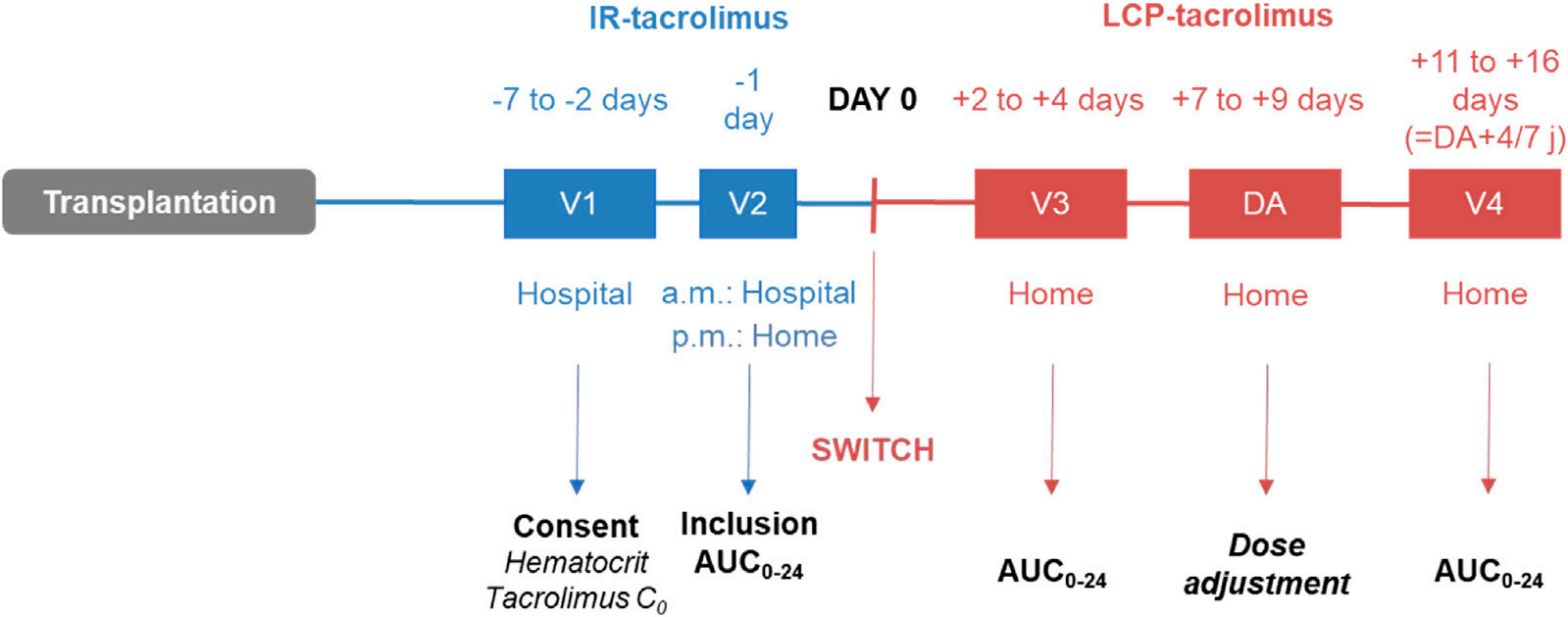
ENVARSWITCH study design.

After inclusion, real-time Bayesian estimation of AUC_0–24 h_ was performed (Figure 1): on the day before the switch (V2), after the IR-tacrolimus morning and evening doses (two AUC_0–12 h_ estimations); 2–4 days after the switch (V3); 7–14 days after V3 (V4). Conversion from IR-tacrolimus to LCP-tacrolimus was done on a 1:0.7 (mg:mg) total daily dose basis. Further dose adjustment could be performed between days 7 and 9, according to the AUC_0–24h_ estimated at V3, to target the pre-switch AUC_0–24 h_ calculated by summing the morning and the evening tacrolimus AUC_0–12 h_. AUC_0–24 h_ at V4 was compared to the individual target AUC_0–24 h_ (V2). No standardized AUC_0–24 h_ target was considered for the study.

### Tacrolimus AUC Determination

AUC_0–24 h_ was obtained by Bayesian estimation and a limited sampling strategy (pre-dose then 1 h and 3 h post-dose for IR-tacrolimus; pre-dose and 8 h then 12 h post-dose for LCP-tacrolimus) [1, 8, 10]. DBS were collected on Whatman™ 903 protein saver cards. At V2, the study nurses collected the DBS necessary for the determination of IR-tacrolimus morning AUC_0–12 h_ and trained the patients to collect DBS autonomously. Afterwards, DBS collection was performed at home by the patients. DBS were post-mailed within 24 h after sampling to Limoges University Hospital for centralized analysis. Tacrolimus concentrations were determined using a high performance liquid chromatography–tandem mass spectrometry method on a 4500 AB-Sciex system (Forster City, CA, United States) validated in accordance with the IATDMCT recommendations [22], covering a concentration range of 1–100 μg/L. AUC estimation and the recommended dose were transmitted to the clinicians *via* a dedicated website within 24 h following DBS reception (maximum 5 days).

### Endpoints

The primary endpoint was the Westlake 90%-confidence interval (CI) of the ratio of the dose-adjusted LCP-tacrolimus steady-state AUC_0–24 h_ (V4) over the pre-switch IR-tacrolimus steady-state AUC_0–24 h_ (V2) after log-transformation, in the entire population.

Secondary endpoints were the Westlake 90%-CI of the ratio of AUC_0–24 h_ at V4 over AUC_0–24 h_ at V2 in KTR and in LTR patients and the differences in and ratios of AUC_0–24 h_, C_max_ and C_0_ between V2 and V3 in each subgroup.

Renal function was assessed as serum creatinine (SCr) and glomerular filtration rate estimated using the CKD-EPI equation [23]. For regulatory reasons, whenever missing, the eGFR was estimated from SCr by applying the CKD-EPI equation and considering the individuals as “not Black,” since there was a very high probability for patients to be of Caucasian or North-African ancestry.

*Post hoc* analyses were performed to examine, in the entire population and in each subgroup: 1) the correlation between the theoretical LCP-tacrolimus dose (calculated by applying the 1:0.7 ratio) and the actual dose at V3; 2) the correlation between the LCP-tacrolimus dose proposed after V3 and the actual dose at V4. Doses and exposure indices were also compared between subgroups and periods.

### Adverse Events (AEs)

All AEs occurring between enrollment and the end of the trial were recorded on an ongoing basis, regardless of whether they were related or not to IR-tacrolimus or LCP-tacrolimus. Seriousness was assessed according to ICH E2A [24] and severity (mild, moderate, severe) according to its impact on activities of daily life. The causality to the investigational drug was independently assessed by the investigator and the sponsor (worst causality) at the time of the event. All AEs were coded using the MedDRA dictionary (version 23.0).

### Statistical Analyses

Statistical analyses were performed using R version 4.0 (R Project for Statistical Computing: ^2^). Categorical data are reported as frequencies and percentages, continuous data as means ± standard deviations (SD). Continuous variables were compared between periods using Student paired-t test.

Data were analyzed for the intent-to-treat population (Full Analysis Set, FAS; KTR and LTR, referred to as “the entire population”) and for the per-protocol set (PPS). The FAS comprised included patients who complied with all study visits, while the PPS was restricted to patients of the FAS with no critical protocol deviation. Unless stated, all results are based on the FAS. Safety analyses were based on all included patients.

The comparison of AUC_0–24 h_ between V2 and subsequent visits was based on the mean ratios between log-transformed AUC_0–24 h_ and their Westlake 90%-CI. AUC_0–24 h_ between visits were deemed bioequivalent if the Westlake 90%-CI fell within the 0.90–1.11 range defined by the European Medicine Agency for the bioequivalence of drugs with a narrow therapeutic index [25–28].

The comparison of the exposure indices between the three periods was done by computing Pearson’s coefficient tests, and calculating the mean relative difference and root mean square error (RMSE) of exposure indices at V3 and V4 with respect to those measured at V2.

### Sample Size

It was estimated that 96 patients would demonstrate a mean ratio of 1 [90%CI within 0.90–1.11] between V4 and V2 log-transformed AUC_0–24 h_, with an expected coefficient of variation = 25% for tacrolimus AUC_0–24 h_ and 80% power. Anticipating that 10% patients may not meet the requirements of tacrolimus C_0_ between 4 and 12 μg/L and hematocrit >0.27, and that 20% may drop out (including missing or poor DBS collection or analysis), the total number of patients to enroll was set to 134.

## Results

### Patients

**FIGURE 2:**
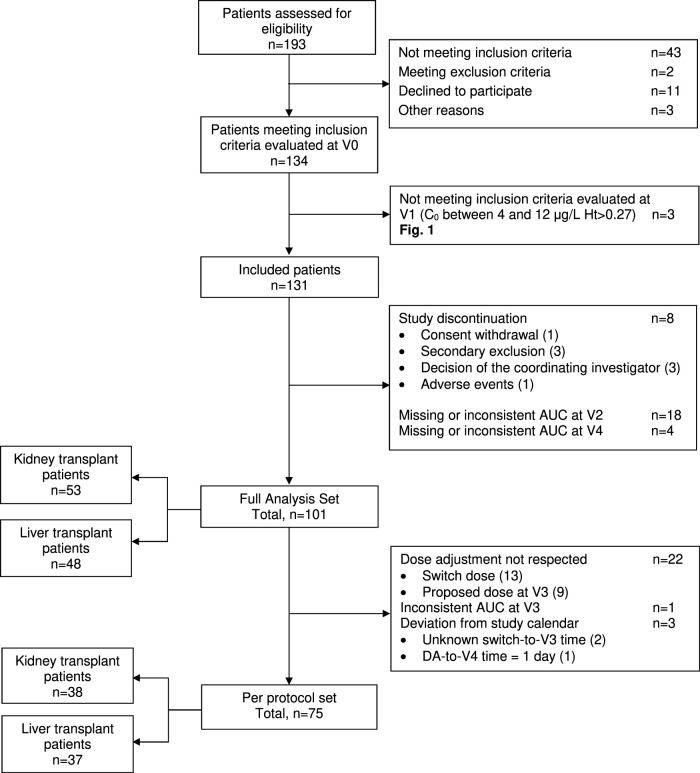
ENVARSWITCH flow diagram following STROBE recommendations.

Overall, 134 patients (70 KTR and 64 LTR) were enrolled. Three patients did not meet the inclusion criteria at V1 and 30 either discontinued study participation or displayed unexploitable AUC_0–24 h_ at V2 (Figure 2). Thus, the FAS comprised 101 patients, of whom 75 constituted the PPS. The KTR and LTR subgroups (Table 1) were comparable in terms of sex ratio, weight, body mass index and haematocrit, but LTR were characterized by a significantly older age (*p* = 0.001), later post-transplantation period (*p* = 0.002), and better kidney function (*p* = 0.022 for SCr and <0.001 for eGFR).

**TABLE 1:** Patient characteristics at V2.

Variables	Full analysis set			Per protocol set		
	Total	Kidney transplant patients	Liver transplant patients	Total	Kidney transplant patients	Liver transplant patients
	*N* = 101	*N* = 53	*N* = 48	*N* = 75	*N* = 38	*N* = 37
Age, years	**53.2 (11.9)**	49.6 (13.2)	57.4 (8.80)	**53.8 (12.0)**	49.0 (13.4)	58.7 (7.93)
Gender (M/F)	**70/31**	32/21	38/10	**52/23**	23/15	29/8
Post-transplantation time, days	**138 (91.8)**	112 (87.7)	168 (87.9)	**137 (90.0)**	107 (83.8)	167 (86.9)
Weight, kg	**74.7 (15.3)**	75.2 (14.3)	74.1 (16.4)	**75.3 (15.8)**	75.9 (14.8)	74.7 (16.9)
Body mass index, kg/m^2^	**25.4 (4.41)**	25.2 (4.01)	25.5 (4.84)	**25.3 (4.64)**	25.1 (4.16)	25.6 (5.11)
Serum creatinine, µmol/L	**118 (51.2)**	129 (32.0)	106 (64.5)	**121 (56.4)**	130 (34.4)	112 (71.8)
eGFR (CKD-EPI), mL/min/1.73 m^2^ ^a^	**62.3 (21.1)**	53.4 (16.1)	72.1 (21.7)	**60.8 (20.4)**	53.6 (17.0)	68.2 (21.2)
Tacrolimus total daily dose, mg	**6.36 (4.12)**	7.75 (4.49)	4.81 (3.03)	**6.03 (3.62)**	7.63 (3.75)	4.39 (2.64)
Tacrolimus C_0_, µg/L	**7.97 (2.01)**	8.58 (1.60)	7.31 (2.21)	**7.90 (1.93)**	8.55 (1.60)	7.26 (2.04)
Hematocrit, %	**37.1 (5.03)**	36.8 (4.85)	37.5 (5.25)	**37.0 (4.97)**	36.8 (5.00)	37.2 (5.00)

Data are presented as mean (SD).

^a^ eGFR considering patients as Caucasians: in FAS, *n* = 35 (22 KTx, 13 LTx); in PPS, *n* = 28 (17 KTx, 11 LTx).

Bold characters are for totals.

### Tacrolimus Dose and Exposure Indices

AUC_0–24_ were distributed normally (Shapiro-Wilk test *p* between 0.067 and 0.2195). At V2, the mean IR-tacrolimus daily dose was significantly higher in KTR than in LTR (*p* < 0.001; Table 2), and so were C_0_ and AUC_0–24 h_ (*p* < 0.001) (Table 1). The difference on daily dose and AUC_0–24 h_ of LCP-tacrolimus between KTR and LTR persisted at V4 (*p* = 0.001 and *p* < 0.001, respectively).

**TABLE 2:** Tacrolimus daily dose (mg/day) and AUC_0–24 h_ (h.µg/L) at each study visit in the full analysis set.

		Total	Kidney transplant patients	Liver transplant patients
		*N* = 101	*N* = 53	*N* = 48
V2	Tacrolimus daily dose	**6.36 (4.12)**	7.75 (4.49)	4.81 (3.03)
Before conversion	AUC_0–24 h_	**229 (77.2)**	266 (70.5)	187 (61.9)
V3	Tacrolimus daily dose	**4.43 (2.87)**	5.51 (3.25)	3.22 (1.73)
After conversion	AUC_0–24 h_	**237 (88.6)**	273 (89.1)	198 (70.3)
V4	Tacrolimus daily dose	**4.48 (3.32)**	5.63 (3.81)	3.22 (2.06)
After dose adjustment	AUC_0–24 h_	**236 (84.0)**	269 (72.2)	200 (82.1)

Data are presented as mean (SD).

Bold characters are for totals.

### Evaluation of the Overall Dose-Conversion and Individual Dose-Adjustment Strategy

**FIGURE 3:**
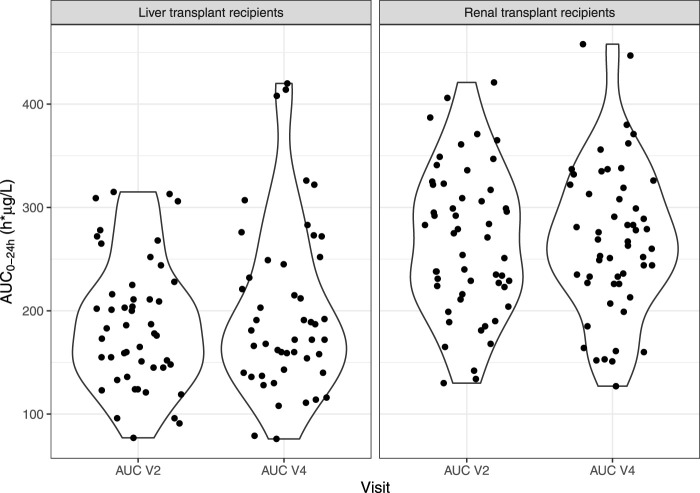
Violin plots of IR-tacrolimus AUC_0–24 h_ (V2) and LCP-tacrolimus AUC_0–24 h_ after dose adjustment (V4) in the full analysis set, split in two subgroups (liver and kidney transplant patients).

The bioequivalence criterion between V2 and V4 was met in the FAS (mean ratio [90% CI] = 1.07 [0.97–1.09]) and the PPS (1.08 [0.97–1.11]). The violin plots of AUC_0–24h_ by subgroup at V2 and V4 in the FAS are presented in Figure 3. No significant difference was observed on the mean AUC_0–24 h_ between V2 and V4 (*p* = 0.297), but correlation was poor (*r* = 0.608), with a mean relative difference between V4 and V2 of 0.074 ± 0.330 h.µg/L and RMSE = 34%.

**FIGURE 4:**
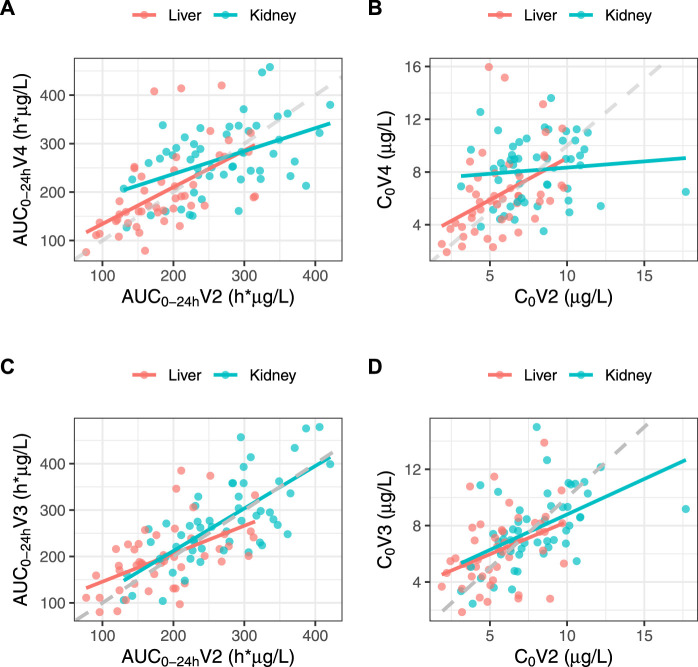
Correlations between IR-tacrolimus (V2) and dose-adjusted LCP-tacrolimus (V4) AUC_0–24 h_ **(A)** and C_0_ **(B)** and between IR-tacrolimus (V2) and dose-converted LCP-tacrolimus (V3) AUC_0–24 h_ **(C)** and C_0_ **(D)** in the full analysis set split in the two transplant subgroups.

The bioequivalence criterion between V2 and V4 was met in KTR (1.05 [0.93–1.09]) and almost met in LTR (1.10 [0.96–1.14]). The correlation between V2 and V4 AUC_0–24h_ was poor in both subgroups (*r* = 0.462 and 0.571, respectively), and even poorer for C_0_ (*r* = 0.100 and 0.429) (Figure 4). No statistically significant C_0_ difference was observed between V2 and V4 for either subgroup (C_0_ = 7.87 ± 2.60 vs. 8.14 ± 2.41, *p* = 0.671 and 5.71 ± 2.12 μg/L vs. 6.33 ± 3.14 μg/L, *p* = 0.150, respectively).

### Evaluation of the Recommended Dose-Conversion Ratio

The bioequivalence criterion between V2 and V3 was met in the entire population (1.06 [0.96–1.08]) and in KTR (1.03 [0.94–1.07]), but not in LTR (1.11 [0.96–1.15]). The correlation between V2 and V3 AUC_0–24 h_ was poor in both subgroups (*r* = 0.724 and 0.531, respectively). Additionally, despite the absence of significant C_0_ differences between V2 and V3 in either subgroup (7.87 ± 2.60 vs. 7.72 ± 2.53 μg/L, *p* = 0.680 and 5.71 ± 2.12 vs. 6.30 ± 2.51 μg/L, *p* = 0.120, respectively), the correlation between V2 and V3 C_0_ was poorer than that of the AUC_0–24 h_ (*r* = 0.516 and 0.391, respectively) (Figure 4). As expected, the mean C_max_ was significantly lower at V3 than at V2 in both subgroups (15.6 ± 5.60 vs. 22.1 ± 9.42 μg/L, *p* < 0.001 and 11.4 ± 3.99 vs. 16.1 ± 6.69 μg/L, *p* < 0.001, respectively).

### Compliance With the Recommended Dose

**FIGURE 5:**
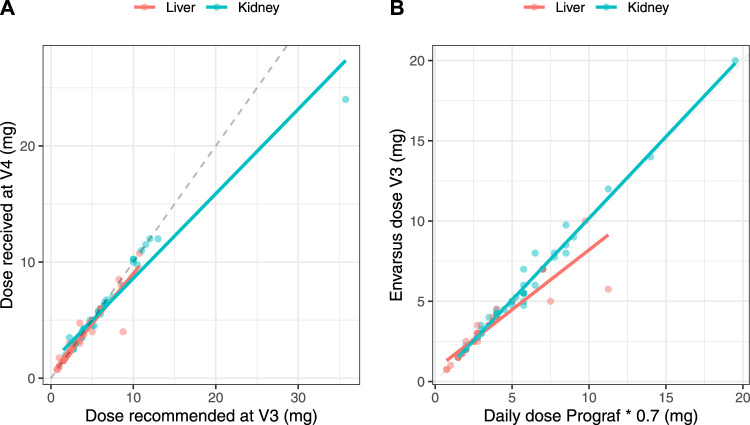
Correlations between the theoretical converted dose (IR-tacrolimus daily dose × 0.7) and the actual dose received by the patient at V3 **(A)** and between the LCP-tacrolimus dose proposed based on the AUC_0–24 h_ at V3 and the dose actually received at V4 **(B)** in the full analysis set split in the two transplant subgroups.

Correlations between the IR-tacrolimus dose at V2 × 0.7 and the LCP-tacrolimus dose at V3, and between the LCP-tacrolimus dose proposed at V3 and the administered dose at V4 were strong in both subgroups (*r* > 0.9, Figure 5), showing overall good compliance of the clinicians with the doses recommended at all steps.

### Impact of Dose Adjustment on AUC_0–24 h_

**FIGURE 6:**
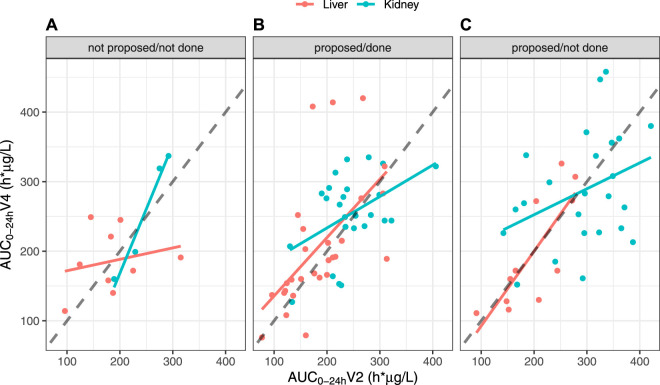
Correlations between AUC_0–24 h_ values at V4 and V2, split by organ and depending on the scenario, in the FAS: **(A)** Dose adjustment after V3 not proposed and not done; **(B)** Dose adjustment after V3 proposed and done; **(C)** Dose adjustment after V3 proposed but not done.

The impact of dose adjustment on AUC_0–24h_ was evaluated by comparing AUC_0–24 h_ V4 vs. V2 depending on whether the patients needed dose adjustment after V3 (88 patients) or not (13 patients) and whether dose adjustment was done (53 patients) or not (35 patients) (Figure 6). No patient benefited from a dose adjustment if no dose adjustment had been proposed. The AUC_0–24 h_ at V4 and V2 were well correlated in KTR who did not require and did not have a dose adjustment (*r* = 0.982), but not in LTR in the same situation (*r* = 0.225). In contrast, among patients for whom we proposed dose adjustment, the correlation was poor in KTR, whether dose adjustment had been applied or not (*r* = 0.458 and 0.356, respectively) and fair in LTR recipients (*r* = 0.581 and 0.794, respectively).

### Renal Function

No difference in renal function was found between V2 and V4 in the entire population (Scr at V4 = 119 ± 50.2 μmol/L, *p* = 0.826; eGFR = 62.5 ± 22.3 mL/min, *p* = 0.974), nor in subgroups separately. The average SCr and eGFR at V4 were, respectively 130 ± 30.0 μmol/L (*p* = 0.953) and 53.4 ± 16.0 mL/min (*p* = 0.708) in KTR and 107 ± 63.0 μmol/L (*p* = 0.780) and 72.4 ± 24.0 mL/min (*p* = 0.818) in LTR.

### Safety

The safety analysis set comprised the 134 patients enrolled (Table 3). Patients were on IR-tacrolimus for a maximum of 7 days and on LCP-tacrolimus for a maximum of 3 weeks during their participation in the study. Nineteen and fifty AEs occurred respectively while on IR-tacrolimus and LCP-tacrolimus. Eleven patients (8.2%) experienced at least one AE while on IR-tacrolimus and 33 (25.8%) while on LCP-tacrolimus. One patient (0.7%) while on IR-tacrolimus and twelve (9.4%) while on LCP-tacrolimus experienced at least one AE considered as possibly related to tacrolimus. The majority of AEs were of mild-to-moderate severity (100% on IR-tacrolimus and 94% on LCP-tacrolimus). The incidence of tremor, diarrhea, and hyperglycemia on LCP-tacrolimus was respectively 3.1%, 1.6%, and 0.8%.

**TABLE 3:** Incidence of adverse events (AE) by system organ class and for each treatment in the safety analysis set.

		IR-tacrolimus		LCP-tacrolimus		
		AEs *n* (%)	Patients *n* ^a^ (%)	AEs *n* (%)		Patients *n* ^a^ (%)
		*N* = 19	*N* = 134	*N* = 50		*N* = 128
Number of patients with at least one AE			11 (8.2)			33 (23.8)
Number of patients with at least one serious AEs		0	0	3 (6.0)		3 (2.3)
Severity						
Mild		12 (63.2)	8 (6.0)	24 (48.0)		18 (14.1)
Moderate		7 (36.8)	4 (3.0)	23 (46.0)		16 (12.5)
Severe		0	0	3 (6.0)		3 (2.3)
MedDRA classification (System Organ Class and Preferred Terms)^b^						
Blood and Lymphatic System Disorders						
Anemia		1 (5.3)	1 (0.7)	1 (2.0)		1 (0.8)
Bicytopenia		—	—	1 (2.0)		1 (0.8)
Leukopenia		2 (10.5)	2 (1.5)	—		—
Lymphopenia		—	—	1 (2.0)		1 (0.8)
Neutropenia		1 (5.3)	1 (0.7)	—		—
Eye Disorders						
Retinal detachment		—	—	1 (2.0)		1 (0.8)
Gastrointestinal Disorders						
Abdominal pain		—	—	2 (4.0)		2 (1.6)
Constipation		1 (5.3)	1 (0.7)	—		—
Diarrhea		—	—	2 (4.0)		2 (1.6)
Dyspepsia		1 (5.3)	1 (0.7)	1 (2.0)		1 (0.8)
Gastrointestinal motility disorder		—		1 (2.0)		1 (0.8)
Gastroesophageal reflux disease		1 (5.3)	1 (0.7)	—		—
Hemorrhoids		1 (5.3)	1 (0.7)	—		—
Mucous stools		—	—	1 (2.0)		1 (0.8)
General Disorders and Administration Site Conditions						
Fatigue		2 (10.5)	2 (1.5)	1 (2.0)		1 (0.8)
Edema peripheral		1 (5.3)	1 (0.7)	—		—
Hepatobiliary Disorders						
Jaundice		—	—	1 (2.0)		1 (0.8)
				(severe)		
Infections and Infestations						
Cytomegalovirus gastrointestinal infection		—	—	1 (2.0)		1 (0.8)
Influenza		—	—	1 (2.0)		1 (0.8)
Sinusitis		—	—	1 (2.0)		1 (0.8)
Investigations						
Alanine aminotransferase increased		—	—	1 (2.0)		1 (0.8)
BK polyomavirus test positive		—	—	1 (2.0)		1 (0.8)
Blood creatinine increased		1 (5.3)	1 (0.7)	3 (6.0)		3 (2.3)
Blood phosphorus decreased		2 (10.5)	2 (1.5)	—		—
Immunosuppressant drug level decreased		—	—	1 (2.0)		1 (0.8)
				(severe)		
Immunosuppressant drug level increased		—	—	2 (4.0)		2 (1.6)
Metabolism and Nutrition Disorders						
Hypercalcemia		1 (5.3)	1 (0.7)	—		—
Hyperglycemia		—	—	1 (2.0)		1 (0.8)
Iron deficiency		—	—	1 (2.0)		1 (0.8)
Vitamin D deficiency		1 (5.3)	1 (0.7)	—		—
Musculoskeletal and Connective Tissue Disorders						
Back pain		1 (5.3)	1 (0.7)	—		—
Tendonitis		—	—	1 (2.0)		1 (0.8)
Nervous System Disorders						
Headache		—	—	1 (2.0)		1 (0.8)
Neuropathy peripheral		—	—	1 (2.0)		1 (0.8)
Sciatica		—	—	1 (2.0)		1 (0.8)
Tremor		1 (5.3)	1 (0.7)	4 (8.0)		4 (3.1)
				(1 severe)		
Psychiatric Disorders						
Anxiety		—	—	1 (2.0)		1 (0.8)
Irritability		—	—	1 (2.0)		1 (0.8)
Nightmare		—	—	1 (2.0)		1 (0.8)
Renal and Urinary Disorders						
Hematuria		—	—	1 (2.0)		1 (0.8)
Pollakiuria		—	—	1 (2.0)		1 (0.8)
Renal failure		—	—	1 (2.0)		1 (0.8)
Urine abnormality		—	—	1 (2.0)		1 (0.8)
Reproductive System and Breast Disorders						
Testicular swelling		—	—	1 (2.0)		1 (0.8)
Respiratory, Thoracic and Mediastinal Disorders						
Cough		—	—	1 (2.0)		1 (0.8)
Lung disorder		—	—	1 (2.0)		1 (0.8)
Wheezing		—	—	1 (2.0)		1 (0.8)
Skin and Subcutaneous Tissue Disorders						
Pruritus		—	—	2 (4.0)		2 (1.6)
Surgical and Medical Procedures						
Eventration repair		—	—	1 (2.0)		1 (0.8)
Vascular Disorders						
Blood pressure inadequately controlled		—	—	1 (2.0)		1 (0.8)
Hot flush		—	—	1 (2.0)		1 (0.8)
Hypotension		1 (5.3)	1 (0.7)	—		—
Total		**19**		**50**		

^a^ Patients with ≥2 AEs in the same preferred term are counted only once for that preferred term.

^b^ MedDRA version 23.0.

Bold characters are for totals.

Three serious AEs occurred in three patients, among which two were related to tacrolimus: one pneumopathy and one sub-therapeutic dosage. This latter AE occurred in a patient who had subtotal colectomy, resulting in a decreased AUC_0-24_ at V3 confirmed by a low C_0_ value, explained by the lower absorption of tacrolimus in its extended-release formulation. The patient was switched back to IR-tacrolimus and excluded from the study.

## Discussion

ENVARSWITCH confirms bioequivalent exposure to tacrolimus in terms of AUC_0–24 h_ in 101 stable KTR or LTR converted from IR-tacrolimus to LCP-tacrolimus using a 1:0.7 dose ratio. It is the first clinical study proposing the combination of DBS and Bayesian estimation for tacrolimus AUC_0–24 h_ determination and dose adjustment. The Westlake interval in the entire population fell within the bioequivalence criteria for narrow therapeutic index drugs [26–28], and no significant difference was found between the mean AUC_0–24 h_ before the switch and after the switch followed by individual dose adjustment. Despite the removal of 30/131 (23%) patients from the FAS, the study remained sufficiently powered to validate its primary objective (N > 96 patients). The higher than expected proportion of drop-outs was compensated by the lower than expected proportion of patients not meeting the inclusion criteria at V2 (hence not eligible for formulation switching). Also, although the analysis was less powered, the Westlake interval calculated from data of the PPS still fulfilled the bioequivalence criteria. These results suggest that the 1:0.7 dose conversion ratio combined with individual dose adjustment is overall adapted. Importantly, as patients’ ethnicity was not collected for regulatory reasons, and because the 1:0.7 conversion factor is not recommended for patients of African origin, we hypothesize that the patients to whom this study was proposed by their treating physician were of other origins, and mostly white Europeans.

The Westlake interval between the AUC_0–24 h_ measured before and right after the switch also fell within the bioequivalence criteria in the entire population. This suggests that before any individual dose adjustment, the 1:0.7 dose conversion ratio is adapted, as proposed from the conversion studies [2–4]. Nevertheless, while subgroup analyses found no difference between AUC_0–24 h_ at V2 and at V3 and V4 in KTR recipients, the Westlake interval was close to, but did not fall within, the bioequivalence criteria in LTR. This might partly be due by a lack of power in the subgroup analysis. Additionally, the correlation between the theoretical and actual doses in both contexts of the conversion and dose adjustment proposal was better in KTR than in LTR (Figure 5). More precisely, the LTR group tended to receive lower doses than those they were supposed to receive, especially for theoretical doses above 5 mg/day, while KTR overall received the theoretical doses. This lack of compliance may partly explain why the Westlake interval did not meet the bioequivalence criteria for narrow therapeutic index drugs in LTR. Still, the correlation between AUC_0–24 h_ at V4 and at V2 in LTR who did not need and did not have dose adjustment after the conversion was poorer than that observed in KTR in the same situation (Figure 6). This observation clearly suggests that the 1:0.7 dose conversion ratio is adequate for KTR patients overall but may need to be slightly decreased and followed by dose adjustment in LTR patients. Still, the Westlake interval fell within the larger acceptance interval [0.8–1.25] recommended by the FDA for bioequivalence studies [29].

The poor correlation between AUC_0–24 h_ at V2 and at the subsequent visits confirms the wide intra-individual variability in tacrolimus exposure [7], which was unfortunately not compensated for by individual dose adjustment. Given the short time of participation in the study, this variability cannot be attributed to long-term tacrolimus clearance variation observed mainly in patients in the late vs early period after transplantation (after M12 vs. before M1). Correlations between C_0_ values at V2 and the subsequent visits were even poorer. Although a decrease in the intra-individual variability of tacrolimus exposure on LCP-tacrolimus vs. IR-tacrolimus could be expected, studies in solid organ transplantation have reported comparable intra-patient variability on C_0_ [30, 31]. Only one study has reported a significantly lower intra-patient variability of the AUC on LCP-tacrolimus (10.9%) vs. IR-tacrolimus (14.1%) [32]. In any case, the poorer correlation between C_0_ values is in favour of considering the AUC_0–24 h_ rather than C_0_, at least when patients are converted from IR-to LCP-tacrolimus, then at regular time points during follow-up.

The poor correlation between AUC_0–24 h_ values before the switch and afterwards may also be due to the relatively short time period between the switch and the subsequent AUC_0–24 h_ measurement. It was ≤3 days in 85/101 patients, so that V3 AUC_0–24 h_ may not reflect steady-state. This may have led to imprecise or even wrong dose recommendations. Furthermore, steady state may not even have been reached at V4 in all patients, as suggested by the poor correlation between AUC_0–24 h_ at V2 and V4 in LTR who did not need and did not have tacrolimus dose adjustment (Figure 6).

Variability may have also come from the use of DBS collected using non-volumetric devices and from the study design, where nurses collected the morning AUC at V2 while the other AUCs were collected by the patients. A comparison of AUC at V3 vs. V4, all sampled by the patients themselves, confirmed the high intra-individual variability (data not shown), dwarfing inter-operator differences as a source of variability. At the time the study was launched, analytical validation data for the measurement of tacrolimus concentrations were available only for the Whatman™ 903 protein saver cards [14, 17, 33]. In the meantime, experience has shown that the insufficient standardization of the volume of blood drops contributes to a relative imprecision of concentration measurements [22]. Another potential source of imprecision could have been the hematocrit (varying between 26.2% and 47.0% among patients at V2), as no correction of the analytical results was performed based on the hematocrit. Various patient-centered volumetric micro-sampling devices are now favored for the TDM of immunosuppressants [15, 16, 22, 34].

Twenty-two patients (17%) were withdrawn from the FAS because of unexploitable AUCs, mostly due to non-compliance with sampling times or poor quality of the DBS samples. Yet, training and a user manual had been provided to the healthcare teams and patients. This suggests that using home-based collection of microsamples requires may require even more training for certain patients, in order for them to understand the importance of respecting the sampling schedule, rigorously collect sampling information, and proceed to proper sample collection.

Interestingly, significantly lower exposure was observed in LTR compared to KTR. This may be related to the large C_0_ target window at inclusion, allowing liver transplant physicians to target lower C_0_ than kidney transplant doctors. This hypothesis is confirmed by the lower daily doses received by LTR compared to KTR (Table 2; *p* < 0.001). As the individual AUC_0–24 h_ at V2 was used as a target for LCP-tacrolimus dose adjustment after V3, the lower exposure in LTR compared to KTR was carried forward throughout the study. Of note, no AUC target has been validated so far for either kidney or liver transplant patients in late periods after transplantation; the only proposed AUC_0–12 h_ target of 150 h.μg/L [7], was derived from a study performed in 100 kidney transplant patients in the early post-transplantation period [35].

The ENVARSWITCH study used an original approach, where the Westlake interval served to compare the mean exposure obtained with the twice daily IR-tacrolimus vs the once daily LCP-tacrolimus formulation at a 0.7 dose ratio and after dose adjustment. The Westlake interval is generally used in bioavailability studies comparing generic to reference formulations, or newer to reference formulations of brand name drugs for instance. The results obtained here allow for the conclusion that LCP-tacrolimus and IR-tacrolimus had bioequivalent AUCs (since C_max_ and T_max_ were not studied), despite the above-mentioned sources of intra-individual variability. This is consistent with previous studies showing that respecting the 1:0.7 dose ratio obviated the need for dose adjustment in the majority of patients [30]. This means that the IR-tacrolimus to LCP-tacrolimus 1:0.7 conversion dose ratio is appropriate on average, in particular in KTR, but may deserve to be refined in LTR. We calculated that the dose ratio that would zero-in the Westlake interval within the acceptance limits for LTR should be 5% lower, i.e., 1:0.665. However, given the small difference and the impossibility of giving each patient a very precise dose, this option was not considered. The best recommendation would therefore be that tacrolimus exposure should be closely monitored in LTR, preferably based on the AUC_0–24 h_, in order to adjust their dose individually, i.e., to compensate for the largest individual exposure differences.

Finally, a relatively low incidence of adverse events was reported in the ENVARSWITCH study. This is related to the short duration of patient participation in the study, especially while on IR-tacrolimus (mean of 6 days between inclusion at V1 and the switch to LCP-tacrolimus).

In conclusion, while the design of the ENVARSWITCH study does not allow comparing therapeutic drug monitoring strategies (AUC or C_0_ monitoring from venous blood or DBS samples), its results suggest that the combination of DBS and Bayesian estimation for tacrolimus dose adjustment elicits reaching rapidly appropriate exposure to tacrolimus after the switch from IR-tacrolimus to LCP-tacrolimus. The use of volumetric microsampling devices should further improve the reliability of AUC_0–24 h_ estimation and individual dose adjustment.

## Data Availability

The raw data supporting the conclusion of this article will be made available by the authors, without undue reservation.

## Footnotes

1
https://abis.chu-limoges.fr/

2
http://www.r-project.org

## Abbreviations

AE, adverse event; AUC, area under the curve; BID, twice a day; C_0_, trough concentration; CI, confidence interval; CKD-EPI, Chronic Kidney Disease EPIdemiology collaboration; C_max_, maximal concentration after drug intake; DBS, dried blood spot; eGFR, estimated glomerular filtration rate; FAS, full analysis set; ICH, International Council for Harmonisation of Technical Requirements for Pharmaceuticals for Human Use; IR-tacrolimus, immediate-release tacrolimus; ISBA expert system, ImmunoSuppressive Bayesian dose Adjustment expert system; KTR, Kidney transplant recipient; LCP-tacrolimus, extended-release tacrolimus; LTR, Liver transplant recipient; MedDRA, Medical Dictionary for Regulatory Activities; PPS, per-protocol set; QD, once a day; RMSE, root mean square error; SCr, serum creatinine; SD, standard deviation; TDM, therapeutic drug monitoring; V, visit.
